# Growth rate of an apical left ventricular myxoma using serial two dimensional echocardiographic and computed tomography observations over twelve months: a case report

**DOI:** 10.1186/1752-1947-8-60

**Published:** 2014-02-19

**Authors:** Panagiota Kourkoveli, Dimitrios Tsiapras, Evaggelia Grisbolaki, Olga Karapanagiotou, Stamatina Kampanarou, Stamatis Kyrzopoulos, Loukas Kaklamanis, Mazen Khoury, Vassilis Voudris

**Affiliations:** 1 Department of Cardiology, Onassis Cardiac Surgery Center, 8 Iasiou Street, 11521 Athens, Greece; 2 Second Department of Cardiac Surgery, Onassis Cardiac Surgery Center, Athens, Greece; 3 Radiology Department, Onassis Cardiac Surgery Center, Athens, Greece

## Abstract

**Introduction:**

Myxomas are the most common benign tumors of the heart. They vary widely in size, and little is known about their growth rate. The present case report is, we believe the first in bibliography that provides images of an apical left ventricular myxoma from transthoracic echocardiography and computed tomography scans taken a year apart.

**Case presentation:**

We present the rare case of a 65-year-old asymptomatic Caucasian man with an apical left ventricular myxoma that grew over a 12-month period. Our patient underwent successful surgical excision of the tumor and had an uneventful postsurgical recovery period.

**Conclusions:**

Left ventricular myxomas are benign and curable tumors. They do not usually present with systemic symptomatology and most of them are diagnosed as sequelae of syncope caused by left ventricular outflow tract obstruction or systemic embolization. Because they are usually removed after diagnosis, the growth rate of myxomas is generally unknown. We present a rare case of the asymptomatic presentation of a left ventricular myxoma and its documented growth during a 12-month period.

## Introduction

Left ventricular (LV) myxomas are more common in women and may arise from any region of the left ventricle, in contrast to atrial myxomas, which usually originate from the interatrial septum. Whereas atrial myxomas are usually associated with symptoms and signs of systemic illness (weight loss, fatigue, fever, anemia, leukocytosis) [1], these symptoms are usually not observed in patients with ventricular myxomas. Although the number of asymptomatic cardiac tumors being detected has increased due to the widespread use of echocardiography, left ventricular myxomas are still rare and the diagnosis is often preceded by syncope caused by left ventricular outflow tract obstruction or systemic embolization. From 1957 to present, 98 cases of LV myxoma have been reported [2]. A case report described by Artel *et al.*[1] demonstrated that LV myxomas can grow, with a rate of increasing volume and diameter that is similar to that of atrial myxomas.

The exact etiology of myxomas is unknown and most cases are sporadic. Familial atrial myxomas have an autosomal-dominant transmission, but these account for <10% of the total. Although familial myxomas may be transmitted without any associated disorders, they may present as a component of a Carney complex, an autosomal-dominant condition comprising myxomas at various sites, endocrine tumors, and spotty pigmentation of the skin. It is well accepted that myxomas can develop after cardiac trauma and that radiofrequency ablation for paroxysmal atrial fibrillation (PAF) increases the risk of thrombus or endocarditis in the atrium [3,4].

Because myxomas are usually removed once the diagnosis is made, their growth rate is generally unknown. The reported growth rates of left atrial myxomas from previous reports vary from no growth to between 1.3 to 6.9mm/month in diameter [5,6]. These estimated growth rates are primarily assumed to be based on linear growth and have been calculated from echocardiography images in case reports of patients with left atrial myxomas that were found either accidentally or after the onset of symptoms and already had echocardiographic follow-up for other reasons.

Although recurrence has been reported, LV myxomas are usually benign and curable [7-9]. The overall recurrence rate of resected myxomas is very low and increases linearly during the first four years after resection. Evidence suggests that recurrence is related to age, positive family history, multifocal localization and to the method of surgical excision [9]. To prevent recurrence, it is necessary to select the best surgical approach that enables adequate visualization of the myxoma and its stalk, and to resect the tumor and underlying myocardium *en masse*.

The surgical approach to ventricular myxomas tends to be site-specific and efforts have been made to improve procedural outcomes and minimize the potential postoperative complications [10]. Minimally invasive excisions as well as atrial and aortic approaches have been successfully used depending on tumor topography. In our patient, ventriculotomy was chosen as the optimal surgical approach after taking into consideration the tumor’s location.

## Case presentation

A 65-year-old Caucasian man with a history of PAF was referred to our hospital for further evaluation of a mass located in the apex of his left ventricle. The mass was discovered incidentally after a transthoracic echocardiographic (TTE) examination during hospitalization for paroxysmal atrial fibrillation.

Our patient’s medical history was unremarkable apart from an unsuccessful pulmonary vein ablation for PAF, which took place 12 months previously. On admission, our patient had a heart rate of 80 beats/minute in sinus rhythm, blood pressure of 130/68mmHg, normal carotid pulse, and absence of murmurs or extra-cardiac sounds. A physical examination was normal and our patient was asymptomatic.

**Figure 1:**
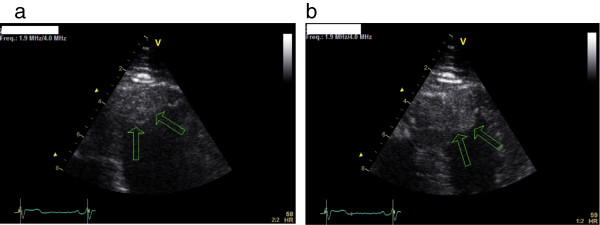
**Transthoracic echocardiography.** Apical two-chamber view in **(a)** systole and **(b)** diastole. A large pedunculated mass located at the apical interventricular septum is seen (arrows). V is a marker which shows where the transducer is.

**Figure 2:**
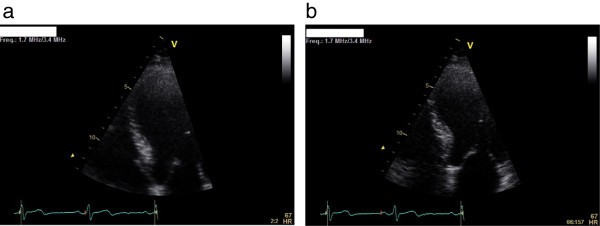
**Transthoracic echocardiography.** Apical two-chamber view in **(a)** systole and **(b)** diastole. No evidence of mass is seen in this view from 12 months previously. V is a marker which shows where the transducer is.

**Figure 3:**
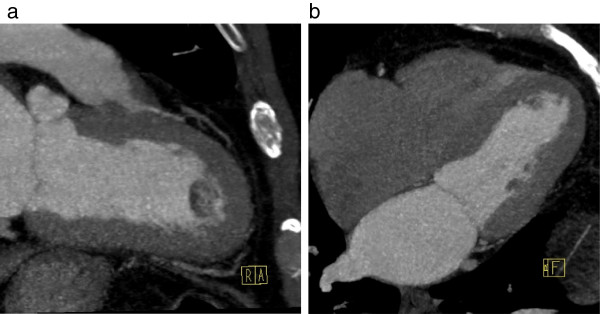
**Computed tomography. (a)** Two-chamber and **(b)** four-chamber axial view images revealing a nodular mass at the apical interventricular septum of the left ventricle. R indicates Right, A anterior and F Foot.

**Figure 4:**
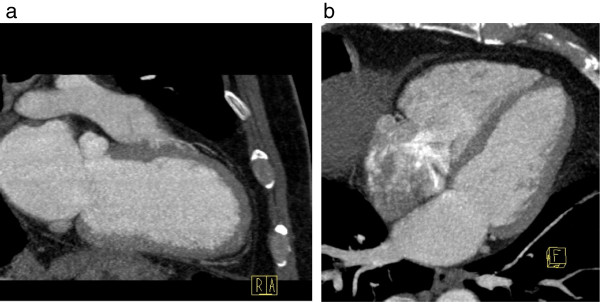
**Computed tomography. (a)** Two-chamber and **(b)** four-chamber axial view images performed 12 months prior to clinical attendance. No mass is seen. R indicates Right, A anterior and F Foot.

A two-dimensional TTE was performed, which showed a large, pedunculated, mobile mass sized 15 × 18mm located at the apical interventricular septum of his left ventricle, with a normally contracting adjacent myocardium (Figure 1a,b). His left ventricular systolic function and the morphology and function of his cardiac valves were normal. His previous TTE performed 12 months previously at the same laboratory and by the same sonographer did not demonstrate any left ventricular mass (Figure 2a,b). Further evaluation with cardiac computed tomography (CT) confirmed the presence of a nodular pedunculated mass (14 × 10mm) with radiographic evidence of myxoma (Figure 3a,b) that was not evident in the examination performed 12 months earlier (Figure 4a,b). The previous TTE and chest CT were performed before our patient underwent pulmonary vein ablation. Magnetic resonance imaging showed a nodular mass of 15mm with a 5mm stalk located at the anterior apical segment of his intraventricular septum. The intensity of the mass was higher than that of the myocardium, with no signs of infiltration of the endocardium.

**Figure 5:**
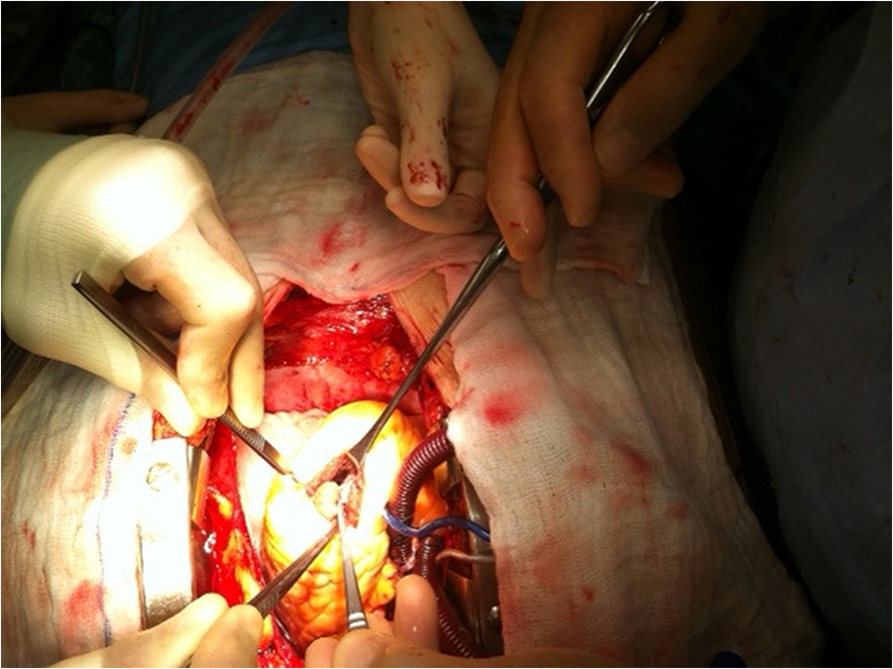
Intraoperative photo showing the mass through an apical left ventriculotomy.

**Figure 6:**
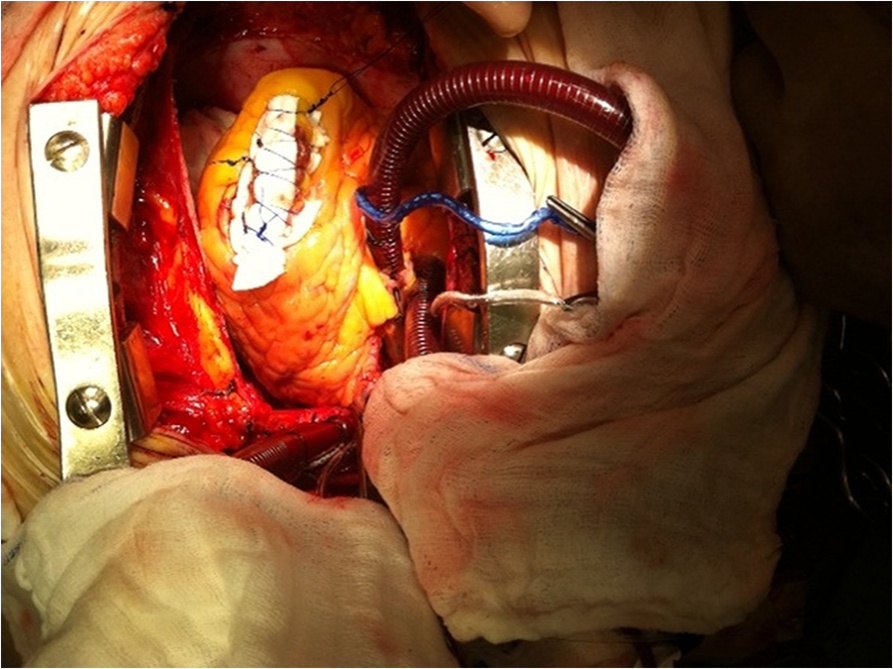
Intraoperative photo of the defect closure.

Our patient underwent urgent cardiac surgery. Under cardiopulmonary bypass and cardioplegic protection, a left apical 2.5cm longitudinal ventriculotomy was performed, which exposed the mass in full view (Figure 5). The defect was closed in a linear fashion and reinforced with felt strips (Figure 6).

**Figure 7:**
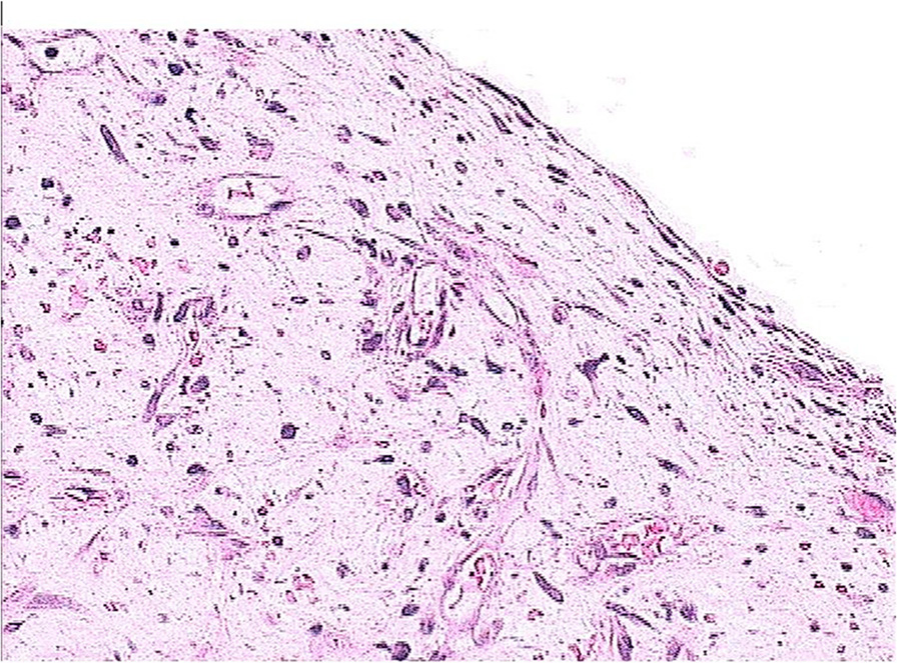
Histopathology of the excised mass shows myxoid stroma containing single and small groups of lepidic cells and a few small vessels.

Resection of the tumor was performed through the ventriculotomy with macroscopically uninvolved margins. A pathology examination demonstrated the typical features of a myxoma (Figure 7). Apart from two self-terminated episodes of PAF, our patient had an uneventful postoperative course and was discharged eight days after the procedure.

Echocardiographic follow-up has not shown evidence of recurrence eight months after the resection of the myxoma.

## Conclusion

Our case represents an unusual location of a cardiac myxoma. Only three cases of apical left ventricular myxoma have been published [1,7,8] and, to the best of our knowledge, this is the first case providing images of an apical left ventricular myxoma from TTE and CT scans obtained a year apart, with an estimated growth rate of 1.2mm/month. No correlation can be found between the radiofrequency ablation of the pulmonary valves that our patient had undergone 12 months prior to the diagnosis and the formation of the myxoma.

## Consent

Written informed consent was obtained from the patient for publication of this case report and accompanying images. A copy of the written consent is available for review by the Editor-in-Chief of this journal.

## Abbreviations

CT: Computed tomography; LV: Left ventricular; PAF: Paroxysmal atrial fibrillation; TTE: Transthoracic echocardiography.

## Competing interests

The authors declared that they have no competing interests.

## Authors’ contributions

PK was responsible for the design and writing of the case report. EG contributed to article drafting and editing. DT provided the images of the echocardiography studies of the patient and revised the article. OK provided the images of the computed tomography studies of the patient. SKa was responsible for editing the images of the computed tomography studies. SKy was responsible for editing the images of the echocardiography studies. LK was the pathologist and provided the microscopic image of the tumor. MK was the patient’s surgeon, and provided the intraoperative images. VV was responsible for approving the article. All authors read and approved the final manuscript.
